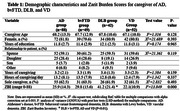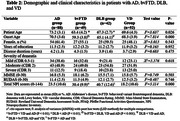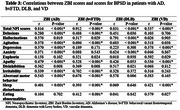# Influence of behavioral and psychological symptoms on caregiver burden for different types of dementia: clinical experience in Lima, Peru

**DOI:** 10.1002/alz.090795

**Published:** 2025-01-03

**Authors:** Rosa Montesinos, Belen Custodio, Marco Malaga, Diego Chambergo‐Michilot, Graciet Verastegui, Katherine Agüero‐Flores, Juan Alejos‐Zirena, Luis Andamayo‐Villalba, Wendy Seminario G, Nilton Custodio

**Affiliations:** ^1^ Unidad de diagnóstico de deterioro cognitivo y prevención de demencia, Instituto Peruano de Neurociencias, Lima, Perú, Lima, Lima Peru; ^2^ Unidad de Investigación, Instituto Peruano de Neurociencias, Lima, Perú, Lima, Lima Peru; ^3^ Cognitive Impairment Diagnosis and Dementia Prevention Unit, Instituto Peruano de Neurociencias, Lima, Perú, Lima, Lima Peru; ^4^ Grupo de Investigación Neurociencia Efectividad Clínica y Salud Pública, Universidad Científica del Sur, Lima, Lima Peru; ^5^ Universidad Científica del Sur, Lima, Perú, Lima, Lima Peru; ^6^ Cognitive Impairment and Dementia Prevention Unit, Peruvian Institute of Neurosciences, Lima, Lima Peru; ^7^ Hospital Sergio Bernales, Lima, Lima Peru; ^8^ Hospital Sabogal, EsSalud, Lima, Lima Peru; ^9^ Hospital IV Augusto Hernández Mendoza‐ EsSalud, Ica, Ica Peru

## Abstract

**Background:**

People caring of individuals with dementia are prone to suffering from burden. Behavioral and psychological symptoms of dementia (BPSD) may have an impact on caregiver burden. In Latin American countries there is lack of research on caregiver burden. We aimed to determine which BPSD have the greatest impact on caregiver burden among Peruvian patients with dementia; and to compare the effects of BPSD on caregiver burden across different types of dementia.

**Methods:**

Cross‐sectional study of 231 patients living with Alzheimer’s dementia (AD), behavioral variant frontotemporal dementia (bvFTD), dementia with Lewy bodies (DLB) and vascular dementia (VD), and their caregivers attended in a Peruvian memory clinic. BPSD were assessed with the Neuropsychiatric Inventory (NPI). Caregiver burden was assessed with the Zarit Burden Inventory (ZBI). We used analysis of variance to compare the groups of AD, bvFTD, DLB and VD. Correlations between ZBI and NPI subscale scores were assessed with Spearman’s correlation.

**Results:**

DLB caregivers had significantly higher levels of burden than the other patient groups (P < 0.05), and higher total NPI scores than caregivers for other patient groups (P < 0.05). bvFTD caregivers had significantly higher total NPI scores than AD and VD caregivers (P < 0.05). Hallucinations, aberrant motor behavior, and apathy were the symptoms most significantly correlated with caregiver burden in those caring for DLB, bvFTD, and AD patients, respectively.

**Conclusions:**

Neuropsychiatric symptoms are higher in DLB caregivers. Hallucinations, aberrant motor behavior, and apathy are the main symptoms correlated with burden.